# Obesity, body composition, and prostate cancer

**DOI:** 10.1186/1471-2407-12-23

**Published:** 2012-01-18

**Authors:** Jay H Fowke, Saundra S Motley, Raoul S Concepcion, David F Penson, Daniel A Barocas

**Affiliations:** 1 Department of Medicine, Vanderbilt University Medical Center, Nashville, TN, USA; 2 Department of Surgical Urology, Vanderbilt University Medical Center, Nashville, TN, USA; 3 Urology Associates, Nashville, TN, USA; 4 VA Tennessee Valley Geriatric Research, Education, and Clinical Center (GRECC), Nashville, TN, USA; 5 Vanderbilt University Medical Center, 2525 West End Ave, 6th floor, suite 600, Nashville, TN, 37203, USA

## Abstract

**Background:**

Established risk factors for prostate cancer have not translated to effective prevention or adjuvant care strategies. Several epidemiologic studies suggest greater body adiposity may be a modifiable risk factor for high-grade (Gleason 7, Gleason 8-10) prostate cancer and prostate cancer mortality. However, BMI only approximates body adiposity, and may be confounded by centralized fat deposition or lean body mass in older men. Our objective was to use bioelectric impedance analysis (BIA) to measure body composition and determine the association between prostate cancer and total body fat mass (FM) fat-free mass (FFM), and percent body fat (%BF), and which body composition measure mediated the association between BMI or waist circumference (WC) with prostate cancer.

**Methods:**

The study used a multi-centered recruitment protocol targeting men scheduled for prostate biopsy. Men without prostate cancer at biopsy served as controls (n = 1057). Prostate cancer cases were classified as having Gleason 6 (n = 402), Gleason 7 (n = 272), or Gleason 8-10 (n = 135) cancer. BIA and body size measures were ascertained by trained staff prior to diagnosis, and clinical and comorbidity status were determined by chart review. Analyses utilized multivariable linear and logistic regression.

**Results:**

Body size and composition measures were not significantly associated with low-grade (Gleason 6) prostate cancer. In contrast, BMI, WC, FM, and FFM were associated with an increased risk of Gleason 7 and Gleason 8-10 prostate cancer. Furthermore, BMI and WC were no longer associated with Gleason 8-10 (OR_BMI _= 1.039 (1.000, 1.081), OR_WC _= 1.016 (0.999, 1.033), continuous scales) with control for total body FFM (OR_BMI _= 0.998 (0.946, 1.052), OR_WC _= 0.995 (0.974, 1.017)). Furthermore, increasing FFM remained significantly associated with Gleason 7 (OR_FFM _= 1.030 (1.008, 1.052)) and Gleason 8-10 (OR_FFM _= 1.044 (1.014, 1.074)) after controlling for FM.

**Conclusions:**

Our results suggest that associations between BMI and WC with high-grade prostate cancer are mediated through the measurement of total body FFM. It is unlikely that FFM causes prostate cancer, but instead provides a marker of testosterone or IGF1 activities involved with retaining lean mass as men age.

## Background

Prostate cancer is the leading cancer diagnosis, and the second-leading cause of cancer-related death, among U.S. men [1]. The American Cancer Society estimates over 240,000 new cases will be diagnosed in 2011, with almost 34,000 deaths attributed to the disease [1]. High prevalence and mortality, as well as the long period of time to tumor development, make prostate cancer an attractive target for prevention. However, little is certain about what causes prostate cancer, or the best prevention approach. Established risk factors such as age, African American race, family history of disease, or genetic variants identified from genome wide association studies have not as yet advanced the development of individualized screening and prevention strategies. At present, early-detection and treatment is emphasized, often through prostate-specific antigen (PSA) testing. However, PSA testing does not differentiate between potentially fatal and non-fatal prostate cancer, and the vast majority of men with localized disease diagnosed in the PSA era are treated unnecessarily for non-life-threatening cancers [2]. Furthermore, the U.S. Food and Drug Administration recently concluded that drugs such as finasteride reduce the risk of low-grade cancer but do not have a favorable risk-benefit profile suitable for broad administration [3]. Thus, new strategies are needed to understand the causes of advanced prostate cancer and who may be most at risk.

Obesity research may provide such an opportunity, with several epidemiologic studies reporting that obese men are at greater risk for the diagnosis of advanced stage prostate cancer, disease progression following treatment, or prostate cancer mortality [4]. In contrast, while obesity may lead to a more aggressive cancer, obesity also may lower the risk of low-grade or localized prostate cancer [4,5]. Multiple biological pathways could be involved in either an increase or decrease in prostate cancer risk, including effects the inflammatory response, aromatase expression and shifts in steroid hormone metabolism, and altered insulin sensitivity [6]. Indeed, drugs such as metformin used to treat Type 2 diabetes are also under consideration in prostate cancer treatment [7].

One challenge toward better understanding the relationship between obesity and prostate cancer is how to interpret body size measures across diverse groups of older men. Body mass index (BMI = kg/m^2^) provides the most common estimate of body adiposity in cancer epidemiologic studies. However, BMI is a limited estimator of adipose mass, with recent analyses suggesting up to 50% of men with body adiposity sufficient to be classified as obese are instead classified as non-obese [8,9]. Older men may over-estimate their height [10] such that BMI is underestimated in studies relying on self-reported data. Few prostate cancer research studies measure waist circumference (WC) or waist-hip ratio (WHR), and the role of centralized adiposity independent of BMI in advancing prostate cancer is unclear [11,12]. Indeed, BMI may be as strongly correlated with total fat-free mass (FFM) as with total fat mass (FM) [9], and BMI does not specifically capture the shift toward centralized fat deposition and abnormal glucose-insulin metabolism and dyslipidemia that occurs with aging [13].

Bioelectrical impedance analysis (BIA) provides a feasible and inexpensive approach to estimate body composition in large-scale epidemiologic studies [14]. Though BIA is not a reference measurement, studies comparing body composition measures between BIA and dual-energy X-ray absorptiometry (DXA) show strong correlation (r ≥ 0.80), such that BIA is able to rank-order study participants on FM and FFM. With this rank-ordering, epidemiologic analyses may compare disease risk between participants with low vs. high exposures. However, only one prior study investigated the association between percent body fat (%BF) from BIA against prostate cancer, and found no association [12].

The purpose of this study is to expand the investigation of body composition and prostate cancer by determining the association between prostate cancer and FM, FFM, %BF as estimated by BIA. Further understanding the relationship between FM, FFM, and traditional body size measures such as BMI with prostate cancer risk may unify a diverse research literature, and may provide clues to the most relevant biological pathways linking BMI to prostate cancer progression.

## Methods

### Study design

The Nashville Men's Health Study is a multi-centered, rapid-recruitment protocol to investigate the clinical, genetic, and behavioral determinants of prostate cancer detection, progression, and treatment outcomes. All recruitment and data collection protocols were approved by IRBs at Vanderbilt University and the Tennessee Valley Veteran's Administration. Men referred for prostate biopsy to Vanderbilt University Medical Center, a large community urology practice, and the Tennessee Valley Veterans Administration Medical Center were targeted for recruitment. These urology clinics receive referrals from physicians throughout metro Nashville, TN, and are the primary providers of diagnostic services for urologic disease in the area. Exclusion criteria included age less than 40 years, a prior prostate cancer diagnosis, prior prostate surgery, current androgen supplementation use, or English insufficiency for informed consent. Recruitment activities, biospecimen collection, and body size measurements occurred prior to the prostate biopsy procedure, thus avoiding biases associated with treatment or knowledge of their disease status. Approximately 90% of eligible men approached for recruitment consented to participate.

### Medical chart review

Data abstraction from urology, surgery, and pathology medical reports included PSA test history, the number of prior biopsies, number of prostate cores collected at biopsy leading to recruitment, and prostate size (ml) at biopsy. Biopsy Gleason score was abstracted for participants diagnosed with cancer to define tumor aggressiveness. Prior research suggests the relationship between obesity and prostate cancer is specific to disease stage or aggressiveness, and we classified cases as having Gleason 6, Gleason 7, or Gleason 8-10 prostate cancer. Controls included men identified at biopsy without prostate cancer, prostatic intraepithelial neoplasia, or atypical or suspicious foci. A single pathologist reviewed over 90% of biopsies, and follow-up chart review for 216 prostate cancer cases found that no cases were diagnosed with metastatic disease at recruitment.

### Measurements

All body size measures were obtained at the time of recruitment by trained research staff. Weight (kg) (no shoes, hospital gown) was measured on a calibrated scale, and height (within 0.1 cm) was measured by stadiometer. Body circumferences were measured using an anthropometric tape measure with built-in tension meter (Gullick II) to ensure an even tension was administered to the tape across participants. Waist circumference was measured at the plane across the iliac crest and usually represents the narrowest part of the torso. Hip circumference was measured at the maximum posterior extension of the buttocks. Two measurements at each site are made in rotational order, with a third measurement if the first two differed by more than 1 cm. Waist-to-Hip ratio (WHR) was calculated from the average waist and hip circumference for each participant. Participants also provided the time of their last food and beverage intakes.

Total body FFM, FM, and %BF were measured using a foot-to-foot BIA scale (TBF-310 GS, Tanita Corporation, Arlington Heights, IL). While wearing a hospital robe, participants stand on the BIA platform such that the electrodes are in direct contact with bare feet. Height, sex, fitness category, and age are entered into the device, and a small, imperceptible, electric current (500 μA current at a frequency of 50 kHz) is applied to one leg. The resistance to this current was measured at a contact placed on the opposite leg, and the proprietary software then estimates total body water, FFM, FM, and %BF.

### Statistical analysis

Prior to analysis, we excluded participants reporting finasteride or dutasteride use that may affect prostate cancer detection (n = 165), or with an outlying BIA impedance reading suggesting uncertain quality (impedance > 4 sd from mean, n = 3). Chi-square and Kruskal-Wallis tests do not hold distributional assumptions and were used to compare crude descriptive statistics between cases and controls. Similarly, we used Spearman correlation coefficients with partial adjustment for age to compare the monotonic relationship between body size and body composition measures. Case-control differences in body size and body composition measures were compared within a linear model adjusting for differences in age (continuous), race/ethnicity (white/non-white), family history of prostate cancer (Yes/No), PSA level (continuous), prostate volume (continuous), and current treatment for diabetes (e.g., metformin, insulin, etc.), cardiovascular disease (e.g., anti-hypertensives, calcium channel blockers, etc.), benign prostatic hyperplasia (alpha-1 adrenergic antagonists), or hyperlipidemia (e.g., statins, etc.). A *p*-value of 0.05 or less was considered statistically significant. Multivariable logistic regression was used to calculate odds ratios (OR) and 95% confidence intervals (95% CI) summarizing the association between body measurements with prostate cancer. We analyzed body composition measures as continuous and as categorized variables. BMI was categorized using World Health Organization criteria, while other measures were categorized at the median or quartile values of the control series. This approach allowed us to describe associations across the range of data, compare participants with low vs. higher body size, and to identify dose-response or non-linear trends that may not be evident with a continuous variable.

We used a risk-difference approach to ask if the associations between BMI or other body size measures could be mediated by FM, FFM or %BF. For example, we first calculated an OR for the association between BMI and prostate cancer, then determined if this OR changed after including FM to the logistic regression model. A shift in the OR toward 1.0 after including FM suggested the association between BMI and prostate cancer could be mediated by that body composition factor.

## Results

Controls were approximately the same age as Gleason 6 cases, but 2-5 years younger than Gleason 7 and Gleason 8-10 cases, respectively (Table 1). Controls also had lower blood PSA levels, and a larger prostate size, compared to cases. Gleason 6 cases were most likely to have a family history of prostate cancer. BPH treatment tended to decrease with cancer aggressiveness; however cases and controls did not significantly differ with regard to race/ethnicity or other factors.

**Table 1:** Study population description

	**Controls (n = 1057)**		**Gleason 6 PC (n = 402)**		**Gleason 7 PC (n = 272)**		**Gleason 8-10 PC (n = 135)**		
	**Median**	**25^th^-75^th^**	**Median**	**25^th^-75^th^**	**Median**	**25^th^-75^th^**	**Median**	**25^th^-75^th^**	p*
	
**Age (years)**	64.0	58.0-70.0	64.5	59.0-70.0	66.0	61.0-72.0	69.0	64.0-75.0	< 0.01
**PSA (ng/ml)**	5.0	4.0-6.8	5.0	4.2-6.7	6.0	4.6-8.4	8.0	5.5-16.4	< 0.01
**Prostate Volume (ml)**	45.1	33.3-62.8	37.7	28.2-53.0	33.0	25.8-45.0	35.6	26.1-49.6	< 0.01
	n	%	n	%	n	%	n	%	
	
**Non-white ethnicity**	103	9.7%	50	12.4%	38	13.9%	14	10.3%	0.17
**Family History PC**	215	20.3%	112	27.9%	58	21.3%	17	12.5%	< 0.01
**Diabetes Tx**	149	14.1%	50	12.4%	40	14.7%	22	16.3%	0.68
**CVD Tx**	591	55.9%	244	60.7%	171	62.9%	77	57.0%	0.12
**BPH Tx**	210	19.9%	74	18.4%	44	16.2%	16	11.9%	0.10
**Hyperlipidemia Tx**	436	41.3%	180	44.8%	121	55.5%	60	44.4%	0.54

*P *value from Chi-Square or Kruskal-Wallis test

Table 2 summarizes obesity and body composition measures for cases and controls, adjusted for age and other factors. Compared to controls, Gleason 8-10 prostate cancer cases had significantly higher levels of BMI, FM, and FFM. Similarly, Gleason 7 cases had significantly higher levels of BMI, WC, WHR, and FFM. Body size and composition measures were not significantly associated with Gleason 6 cases. Further adjustment for time since last food or time since last beverage did not affect results.

**Table 2:** Adjusted mean body size and body composition measures

	Controls		Gleason 6 PC		Gleason 7 PC		Gleason 8-10 PC		*p *(vs. control)		
	Mean	95% CI	Mean	95% CI	Mean	95% CI	Mean	95% CI	Gl 6	Gl 7	Gl 8-10
**BMI**	29.6	29.0, 30.1	29.3	28.7, 29.9	30.2	29.5, 30.9	30.5	29.5, 31.4	0.31	0.05	0.04
**WC**	105.2	103.7, 106.6	104.1	102.4, 105.8	107.3	105.4, 109.3	107.3	104.7, 109.9	0.99	0.02	0.09
**WHR**	1.02	1.01, 1.02	1.01	1.00, 1.02	1.03	1.02, 1.04	1.00	1.00, 1.03	0.31	< 0.01	0.68
**FM**	27.1	25.9, 28.4	26.3	24.7, 27.8	28.0	26.3, 29.7	29.9	27.6, 32.2	0.20	0.28	0.01
**FFM**	62.9	62.0, 63.8	63.1	62.0, 64.2	64.4	63.1, 65.6	66.2	64.5, 67.9	0.73	0.01	< 0.01
**%BF**	29.2	28.5, 29.9	28.6	27.7, 29.4	29.4	28.5, 30.4	30.1	28.8, 31.4	0.10	0.64	0.17

Mean values adjusted for age (years), PSA (ng/ml), prostate volume (ml), race (white, non-white), family history (Yes, No/unsure), and current treatment for diabetes, BPH, CVD, or hyperlipidemia

We ran multivariable logistic regression analyses using body size variables on a continuous scale, or after categorization to identify trends or thresholds (Table 3). BMI was marginally associated with Gleason 8-10 prostate cancer (continuous: OR = 1.039 (1.000, 1.081); BMI > 30: OR = 1.45 (0.93, 2.25)) while a BMI ≥ 35 was significantly associated with Gleason 7 cancer (OR = 2.05 (1.19, 1.93)). WC was significantly associated with Gleason 8-10 (Q4: OR = 1.90 (1.03, 3.53)) and Gleason 7 cancer (continuous: OR = 1.012 (1.002, 1.022)). Although there was a significant association between WHR and Gleason 7 cancer, WHR was not associated with Gleason 8-10 cancer. BMI, WC, and WHR were not significantly associated with Gleason 6 prostate cancer.

**Table 3:** Association between body size and body composition with prostate cancer

		Gleason 6 PC			Gleason 7 PC			Gleason 8-10 PC		
Measure	scale	n	OR	95% CI	n	OR	95% CI		OR	95% CI
BMI	per kg/m^2^	402	0.989	0.964, 1.015	272	1.025	0.997, 1.055	135	1.039	1.000,1.081
	< 25	78	1.0	ref	55	1.0	ref	26	1.0	ref
	25-29.9	198	1.17	0.85, 1.61	116	1.12	0.75, 1.64	62	1.63	0.90, 2.91
	30-34.9	97	1.05	0.72, 1.51	66	1.24	0.80, 1.93	37	2.24	1.19, 4.23
	≥ 35.0	29	0.96	0.57, 1.62	35	2.05	1.19, 1.93	10	1.41	0.54, 3.68
	≥ 30 vs. < 30 (ref)	126	0.92	0.71, 1.19	101	1.33	0.98, 1.81	47	1.45	0.93, 2.25
WC	per cm	402	0.994	0.984, 1.004	272	1.012	1.002, 1.022	135	1.016	0.999,1.033
	Q1	99	1.0	ref	66	1.0	ref	33	1.0	ref
	Q2	106	1.07	0.76, 1.49	60	0.98	0.65, 1.49	27	1.22	0.65, 2.29
	Q3	120	1.10	0.79, 1.53	75	1.18	0.79, 1.77	39	1.66	0.92, 2.98
	Q4	77	0.94	0.65, 1.35	70	1.46	0.95, 2.23	36	1.90	1.03, 3.53
	High vs. Low (ref)	197	1.00	0.78, 1.28	145	1.31	0.97, 1.75	75	1.59	1.04, 2.44
WHR	per 0.1 unit	402	0.898	0.756, 1.066	272	1.232	1.029, 1.474	135	1.034	0.769,1.388
	Q1	100	1.0	ref	51	1.0	ref	29	1.0	ref
	Q2	112	0.99	0.71, 1.37	61	0.98	0.63, 1.51	37	1.04	0.57, 1.88
	Q3	85	0.85	0.60, 1.21	79	1.44	0.94, 2.20	24	0.88	0.47, 1.67
	Q4	105	0.86	0.61, 1.21	80	1.25	0.81, 1.92	45	1.11	0.61, 2.00
	High vs. Low (ref)	190	0.86	0.68. 1.10	159	1.36	1.01, 1.83	69	0.98	0.65, 1.49
FM	per kg	402	0.993	0.981, 1.004	271	1.010	0.997, 1.024	135	1.020	1.004,1.037
	Q1	105	1.0	ref	63	1.0	ref	35	1.0	ref
	Q2	111	1.09	0.79, 1.51	67	1.17	0.77, 1.78	24	0.99	0.52, 1.88
	Q3	100	1.00	0.71, 1.39	67	1.34	0.88, 2.05	40	2.18	1.22, 3.87
	Q4	86	0.88	0.62, 1.25	75	1.52	1.00, 2.30	36	1.65	0.90, 3.03
	High vs. Low (ref)	186	0.90	0.71, 1.15	142	1.32	0.98, 1.77	76	1.93	1.25, 2.97
FFM	per kg	402	1.006	0.991, 1.021	271	1.029	1.011, 1.048	135	1.047	1.021,1.074
	Q1	100	1.0	ref	78	1.0	ref	38	1.0	ref
	Q2	96	0.98	0.70, 1.38	63	1.01	0.68, 1.51	25	1.11	0.59, 2.06
	Q3	118	1.42	1.02, 1.99	60	1.16	0.76, 1.76	38	2.42	1.34, 4.39
	Q4	88	1.14	0.80, 1.64	71	1.72	1.14, 2.61	33	2.91	1.56, 5.44
	High vs. Low (ref)	206	1.31	1.02, 1.67	131	1.40	1.04, 1.89	71	2.50	1.60, 3.91
%BF	per%	402	0.985	0.968, 1.004	272	1.002	0.980, 1.025	135	1.017	0.987, 1.048
	Q1	108	1.0	ref	60	1.0	ref	36	1.0	ref
	Q2	109	0.99	0.72, 1.38	68	1.29	0.85, 1.96	26	1.05	0.57, 1.93
	Q3	108	1.04	0.75, 1.44	84	1.63	1.08, 2.45	40	1.65	0.94, 2.92
	Q4	77	0.71	0.50, 1.02	60	1.07	0.70, 1.65	33	1.21	0.66, 2.19
	High vs. Low (ref)	185	0.88	0.69, 1.12	144	1.18	0.88, 1.58	73	1.39	0.92, 2.12

All analyses adjusted for age (years), PSA (ng/ml), prostate volume (ml), race (white, non-white), family history (Yes, No/unsure), and current treatment for diabetes, CVD, BPH, or hyperlipidemia. Q = quartile. High vs Low defined at median value

Similar analyses of body composition measures found the highest quartile of FFM was significantly associated with Gleason 7 (OR = 1.72 (1.14, 2.61)) and Gleason 8-10 (OR = 2.91 (1.56, 5.44)) prostate cancer. While a FFM above the median level was also significantly associated with Gleason 6 cancer (OR = 1.31 (1.02, 1.67)), there was no dose-response trend in the association. FM as a continuous variable was significantly associated with Gleason 8-10 cancer (OR = 1.020 (1.004, 1.037)), while the highest quartile of FM was significantly associated with Gleason 7 cancer (OR = 1.52 (1.00, 2.30)). %BF was not consistently associated with any grade of prostate cancer.

The correlation between body size and composition measures for cases and controls and with partial adjustment for age is summarized in Table 4. The correlation structure was similar across all cancer cases and controls. BMI and WC were strongly correlated with FM and %BF, and more moderately correlated with FFM. The correlation between FFM and FM ranged from 0.49 to 0.62 across cases and controls.

**Table 4:** Partial Spearman correlation coefficients (adjusted for age) between body size and body composition in controls, and Gleason 6, Gleason 7, and Gleason 8-10 prostate cancer cases

		WC	WHR	FM	FFM	%BF
BMI	Control	0.88	0.50	0.90	0.60	0.83
	Gleason 6	0.88	0.56	0.89	0.55	0.82
	Gleason 7	0.91	0.58	0.91	0.71	0.80
	Gleason 8-10	0.82	0.42	0.80	0.53	0.77
WC	Control		0.72	0.89	0.62	0.80
	Gleason 6		0.75	0.88	0.57	0.80
	Gleason 7		0.75	0.89	0.69	0.79
	Gleason 8-10		0.66	0.83	0.62	0.79
WHR	Control			0.51	0.27	0.51
	Gleason 6			0.57	0.21	0.57
	Gleason 7			0.57	0.32	0.54
	Gleason 8-10			0.41	0.27	0.42
FM	Control				0.57	0.93
	Gleason 6				0.50	0.94
	Gleason 7				0.62	0.92
	Gleason 8-10				0.49	0.88
FFM	Control					0.30
	Gleason 6					0.26
	Gleason 7					0.37
	Gleason 8-10					0.30

All *p *< 0.001

In the context of such correlated data, we conducted a risk-difference analyses to identify whether FM, FFM, or%BF mediate the association between prostate cancer and BMI, WC, or WHR. After controlling for FM or FFM, the association between BMI and Gleason 8-10 was reduced from OR = 1.039 (1.000, 1.081) to OR = 1.010 (0.951, 1.073) or OR = 0.998 (0.946, 1.052), respectively. Similarly, the increase in Gleason 8-10 prostate cancer associated with greater WC was lost with control for FM (OR = 1.002 (0.977, 1.027)) or FFM (OR = 0.995 (0.974, 1.017)). Interestingly, controlling for FFM produced the greatest change in the BMI or WC associations with prostate cancer. In contrast, the significant association between WHR and Gleason 7 prostate cancer was not substantially affected by controlling for FM or FFM (OR = 1.203 (1.002, 1.444)); OR = 1.177 (1.000, 1.386), respectively. Controlling for %BF did not substantially reduce any association.

In full models that include FFM and FM together, only FFM remained significantly associated with Gleason 7 (continuous: OR = 1.030 (1.008, 1.052)) or Gleason 8-10 (continuous: OR = 1.044 (1.014, 1.074)); Q4: OR = 2.62 (1.26, 5.45)) prostate cancer (Table 5). FM was not significantly associated with Gleason 7 or Gleason 8-10 prostate cancer after controlling for FFM, although FM was marginally protective for Gleason 6 prostate cancer (OR = 0.987 (0.974, 1.001)) after controlling for FFM.

**Table 5:** Separating the effects of fat mass and fat free mass on prostate cancer

		**Gleason 6 PC**		**Gleason 7 PC**		**Gleason 8-10 PC**	
		**OR**	**95% CI**	**OR**	**95% CI**	**OR**	**95% CI**
		
FM*	kg	0.987	0.974, 1.001	0.999	0.983, 1.015	1.005	0.983, 1.027
FFM	kg	1.015	0.997, 1.033	1.030	1.008, 1.052	1.044	1.014, 1.074
		OR	95% CI	OR	95% CI	OR	95% CI
		
FM**	Q1	1.0	reference	1.0	reference	1.0	reference
	Q2	0.99	0.70, 1.40	1.13	0.73, 1.74	0.79	0.40, 1.56
	Q3	0.86	0.60, 1.24	1.19	0.75, 1.89	1.48	0.76, 2.88
	Q4	0.75	0.50, 1.11	1.24	0.76, 2.01	0.97	0.47, 1.98
FFM	Q1	1.0	reference	1.0	reference	1.0	reference
	Q2	1.03	0.72, 1.46	0.96	0.63, 1.46	1.02	0.52, 1.97
	Q3	1.54	1.07, 2.21	1.07	0.68, 1.69	2.18	1.15, 4.27
	Q4	1.32	0.88, 2.00	1.53	0.94, 2.50	2.62	1.26, 5.45

Table 5 includes analyses that describe the association between FFM or FM on PC after controlling for either FM or FFM. Analyses evaluate FM and FFM as either continuous variables or at quartiles

* PC = FM (continuous), FFM (continuous), age, prostate volume, PSA, race, family history, and current treatment for diabetes, CVD, BPH, and hyperlipidemia

** PC = FM (Quartiles), FFM (Quartiles), age, prostate volume, PSA, race, family history, and current treatment for diabetes, CVD, BPH, and hyperlipidemia

## Discussion

The relevance of interpreting BMI in older men increases as attention focuses accumulating research that obesity advances prostate cancer. Such information serves both to indentify biological pathways in prostate carcinogenesis and toward identifying intervention targets for outcomes research. We utilized BIA as a complementary body measure to better understand the component of BMI that most affects prostate cancer. In this large study of over 800 prostate cancer cases, we found Gleason 7 and Gleason 8-10 prostate cancers were significantly associated with both FM and FFM. Furthermore, FFM remained associated with high-grade prostate cancer after controlling for FM, and FFM appeared to mediate observed associations between BMI, WC, or FM.

Results from our investigation of BMI and WC are consistent with past studies reporting obesity increases the risk of advanced-stage or high-grade prostate cancer [4,5]. Higher BMI and WC (continuous or categorized) were associated with increased risk of Gleason 7 and Gleason 8-10 prostate cancer, although a limited number of men with BMI of 35 or more may have limited observing a clear trend with Gleason 8-10 prostate cancer. WHR, in contrast, was associated with Gleason 7 cancer but not with Gleason 8-10 cancer. Furthermore, BMI, WC, and WHR were not significantly associated with Gleason 6 cancer. Interestingly, the BMI or WC associations were generally stronger for Gleason 8-10 prostate cancer than for Gleason 7 cancer, consistent with promoting a more aggressive phenotype. Obesity increases oxidative stress, estrogen and leptin levels, inflammatory responses, energy availability, and insulin insensitivity, while decreasing adiponectin [6,15]. Thus, observed associations could be reasonably interpreted as deriving from the effects of excess total or centralized body adiposity.

We used BIA to investigate the relationship between body adiposity and prostate cancer. BIA is an accessible and inexpensive body composition method and provides an opportunity to confirm relationship between FM and prostate cancer. However it must be recognized that body composition estimates from BIA do not always equal those derived from DXA or other reference methods [16-18]. Furthermore, the BIA prediction equations are less accurate among persons with either a very high or low BMI, and cannot address differences in hydration or electrolyte levels, or chronic renal failure [17]. This error limits BIA as a quantitative assessment method for many individuals wishing to know their true body composition. Despite these limitations, BIA estimates of body composition are strongly correlated with those from DXA (r ≥ 0.80) [16-19]. In unpublished data, we found strong correlations between foot-to-foot BIA and DXA (FM: r = 0.96, FFM: r = 0.82, %BF: r = 0.85, n = 31 men ages 40-85 years). These studies illustrate that BIA has limitations, but also that BIA is sufficient to rank-order participants, allowing us to ask if those with a higher level of exposure have a different prostate cancer risk than those with a low level.

Contrary to our original hypothesis, we found that FFM best mediated the statistical association between BMI, WC, and FM with high-grade prostate cancer. This does not mean, however, that greater FFM increases the risk of prostate cancer, but instead probably reflects shared genetic, hormonal, or nutritional factors for both the maintenance of lean body mass and prostate carcinogenesis. For example, pituitary growth hormone (GH) induces insulin-like growth factor 1 (IGF1) from the liver and muscle to increase skeletal muscle mass [20]. GH and IGF1 levels decline with aging, concordant with loss of muscle and bone mass and an increase in fat deposition and BMI [21], and selected studies have reported an association between prostate cancer and blood IGF1 [22,23] or genetic variants in *IGF1 *[24] or *IGFBP3 *[25]. Interestingly, the PI3K/AKT/mTOR pathway plays an important role in translating IGF1 signals to protein synthesis and inhibiting muscle degradation and sarcopenia, and this pathway also regulates steroid, protein, ATP, and fatty acid synthesis critical in prostate carcinogenesis [20,26,27]. Alternatively, androgens almost certainly play a role in prostate cancer progression [28], and circulating testosterone levels decline with aging and are associated with centralized fat deposition and loss of lean mass [29,30]. Men with sufficient androgen activity to support lean body mass and control fat deposition in aging may also have increased prostate cancer risk. Indeed, androgen and GH/IGF1 activities may both be involved, such that FFM may reflect the phenotypic marker of cumulative systemic systems involved in prostate carcinogenesis [31]. Interestingly, this does not preclude obesity as a risk factor for high-grade prostate cancer. Many of these effects of obesity operate through the PI3K/ATK/mTOR (e.g., insulin) or LkB-AMPK/mTOR (e.g., adiponectin) pathways [26], such that obesity affects pathways which may, in turn, support FFM retention. We also observed a non-significant protective association between FM and Gleason 6 cancer [5] that became stronger after controlling for FFM, perhaps suggesting that FFM is somewhat obscuring the ability to identify an association between obesity and low-grade prostate cancer.

Strengths of this investigation include a study population with a sufficient number of high-grade cases for analysis. Body size and body composition measures were ascertained by trained staff and prior to diagnosis to prevent reporting error or treatment effects [10]. Although prostate biopsy may miss a cancer, potential bias derived from latent cancer within the control group was minimized because all controls were without cancer at biopsy. BMI is associated with lower blood PSA levels and a larger prostate size, such that potential stage-specific associations between obesity and prostate cancer may be an artifact of factors that influence the ability to detect prostate cancer [32]. However, the study design controls for selective healthcare access, and we controlled for PSA levels, prostate size, and obesity-related diseases that may affect prostate cancer detection or grading.

The study has several limitations. Although it is unlikely that FFM or FM levels are a consequence of a latent or undiagnosed prostate tumor, we cannot say with certainty whether current or past FFM and FM is most relevant. Addressing this would require a prospective study with repeated data collection during follow-up. BIA was chosen as a body composition assessment method because it is feasible for large epidemiologic studies, but as discussed above, BIA is susceptible to certain errors. Our results were not affected by the time between BIA measurement and the participant's last meal or last liquid. We were unable to evaluate the impact of kidney function, but have no reason to believe that kidney function differed between cases and controls. The majority of participants were white, and therefore our results may not generalize to other race/ethnicities. Chance findings can never be entirely ruled out. Replication is necessary to confirm our findings and to extent these results to other race/ethnic groups.

## Conclusions

In summary, multiple body size indices were associated with high-grade prostate cancer. Furthermore, FFM remained significantly associated with high-grade prostate cancer after controlling for FM, BMI, and WC.

## Competing interests

The authors declare that they have no competing interests.

## Authors' contributions

JHF and DAB developed the hypothesis. JHF, RSC, and SSM led data collection, and JHF conducted the statistical analysis and was the primary author. All authors provided comments on earlier drafts. All authors read and approved the final manuscript.

## Pre-publication history

The pre-publication history for this paper can be accessed here:

http://www.biomedcentral.com/1471-2407/12/23/prepub
